# The surprising costs of on-site surgical team CRM training: a Dutch example analyzed

**DOI:** 10.1186/s41077-025-00367-x

**Published:** 2025-07-02

**Authors:** Tessa L. Verhoeff, Jeroen J. H. M. Janssen, A. Eveline Röell, Reinier G. Hoff

**Affiliations:** 1 Department of Anesthesiology, University Medical Center Utrecht, Utrecht University, Q.04.2.313 P.O. Box 85500, Utrecht, 3508 GA The Netherlands; 2 Department of Education, Utrecht University, Utrecht, The Netherlands

**Keywords:** Surgical team, Operating room team, Team training, Costs, Financial aspects, Crew resource management

## Abstract

**Background:**

Interprofessional team training is increasingly implemented in healthcare, especially in the acute care domain. Research shows a positive effect of Crew Resource Management (CRM) training on teamwork and non-technical skills, and there are indications that it might improve patient care. However, CRM training requires a lot of resources, time, and energy. There is a paucity of data on the costs of these programs. The objective of this study was to evaluate and categorize costs related to an in-situ CRM training program for surgical teams in the Netherlands.

**Methods:**

An evaluation of costs was made for an in-situ CRM training program in the operating room (OR) in a tertiary academic center in the Netherlands. The program consisted of 20 half-day training sessions per year. Costs were evaluated for the year 2024. A distinction was made between costs and missed revenues due to not performing elective surgeries.

**Results:**

Total costs of one half-day session added up to roughly €11.700-€15.700,of which 68–76% was due to missed revenues. The other major costs concern salaries of the participants, which made up 12–16% of the total cost of a training session.

**Conclusions:**

In-situ CRM training in the OR is expensive, especially due to missed revenues. These costs need to be transparent to enable healthcare administrators to carefully allocate funds in their institutions. The costs of in-situ team training might balance against possible advantages in training quality due to the use of the actual clinical environment and to potential financial benefits through improved team performance. But this remains as yet unclear. (Quasi-)experimental studies are required to compare simulations on both patient or learner outcomes and financial aspects.

**Supplementary Information:**

The online version contains supplementary material available at 10.1186/s41077-025-00367-x.

## Introduction

Crew resource management (CRM) training was developed in aviation, after several incidents in which human factors played a role. It was adapted to the medical context with the aim of improving non-technical skills (e.g., collaboration, communication and situational awareness) of healthcare professionals. Currently, it can be considered a form of interprofessional team training and is often simulation-based. Although there are indicators that CRM training has positive effects on team functioning [1–3], it is difficult to determine if interprofessional CRM training is “worth our while”, as these types of training require a lot of resources, time and space. Also, during training, participants are not able to perform their “normal” work tasks, thereby limiting their clinical productivity. Information regarding the total costs of these training programs is limited.

CRM training is most frequently part of educational programs in environments such as the emergency department (ED), intensive care unit (ICU), or operating room (OR). CRM training can have a positive impact on perceived communication and teamwork skills of healthcare professionals [1]. Its effect on patient care has also been studied, for example in the ICU, where implementation of interprofessional CRM training and patient safety checklists led to a decrease in complications and mortality [2]. A similar initiative in the ED improved the safety climate, although it also resulted in longer patient stays [3]. In the OR, interprofessional CRM training has been linked to enhanced efficiency [1]. Furthermore, interprofessional teamwork fosters collaboration and cooperation, contributes to greater autonomy, engagement, and job satisfaction among healthcare workers, and decreases their intention to leave their job [4]. Thus, interprofessional CRM training may play a key role in building a healthier, more stable workforce. Therefore, Dutch health authorities have mandated interprofessional team training for surgical teams in several hospitals in the Netherlands in response to the occurrence of major incidents related to lack of communication or collaboration.

Few studies have investigated the financial aspects of (medical) education [5–7]. This could be explained by, among other things, a lack of financial training for educational researchers, difficulty in measuring outcomes of educational research, and lack of demand by education policymakers [5]. However, there are a few studies on the costs of simulation-based team training. Several of these refer to obstetric care [7, 8], next to studies concerning trauma team training [9] and surgical emergencies [10].

In the current study, we focus on financial aspects of in-situ CRM training for surgical teams, as these aspects are largely unknown. Our aim is to provide stakeholders in medical education with insight into the costs of in-situ CRM training.

## Methods

### Setting and participants

In 2021, we started with interprofessional CRM training on the OR at University Medical Center in Utrecht, a tertiary academic health center in the Netherlands. The OR center consists of 24 operating rooms and caters to all surgical specialties.

The program consists of 20 half-day high-fidelity in-situ simulation training sessions each year, performed on 10 days. One session covers a half-day of simulation training in which a complete operating team is trained, i.e., a surgical specialist, two operation assistants, an anesthesia assistant, and an anesthesiologist. The training targeted certified professionals. No residents or trainees participated in the training. A session consists of an introduction and two simulations, each followed by a structured debriefing session. The introduction consisted of a short lecture regarding the importance of CRM training, explaining its principles (such as closed loop communication and situational awareness), and familiarization with the mannequin and the environment. The scenarios used in the simulations varied per surgical specialism. For example, simulations involving Ear, Nose and Throat surgeons included a patient with a difficult airway, and simulations involving cardiothoracic surgeons included a patient with a cardiac tamponade. Each session consisted of two different scenarios, which were of a similar difficulty, as the training was primarily focused on communication and teamwork. The duration of the training sessions and the design of the sessions is in line with previously described CRM training programs in the Netherlands [11]. The training is led by EuSim certified instructors. An internal trainer, either an anesthesiologist or anesthesia assistant, runs the scenario and leads the debriefing session. The hospital hires an external facilitator to aid with running the scenarios and the debriefing session. This facilitator is a trained ICU nurse and paramedic, with several decades of experience in simulation-based education. In each training session, a different OR team was trained, although it was possible that individual professionals participated multiple times. They were scheduled to do so by the OR coordinators instead of a regular OR day. Training was arranged in-situ, because of previously expressed wishes of OR personnel to be able to do their training in their ‘natural surroundings’ with their regular equipment. In this way, the simulation resembled actual clinical circumstances in an optimal fashion.

### Costs

To determine the costs of the training in the year 2024, we applied the method described by Levin [5]. We first identified the resources required for the training. We then evaluated the costs of these resources with the help of the in-house instructors and the external facilitator and the business administration office of the Division of Anesthesiology, Intensive Care and Emergency Medicine of the University Medical Center Utrecht. Costs consisted of average salaries of the participants and instructors, the materials used and of the expenses associated with the OR (including light, ventilation, supply of oxygen, heating, cleaning, etc.). All participants were employed by the hospital. Salaries were determined in accordance with the Dutch collective labor agreement, the “CAO UMC” [12], based on professions and work experience. The average salaries were calculated by to the business administration office. The salary of the external facilitator is an hourly rate for hiring experienced facilitators, and is conform usual rates for such expertise in the Netherlands. The list of used materials during a training was meticulously made in consultation with the training instructors, after which the costs were provided by the hospital administration. In our department, a sufficient number of instructors were already certified and available to organize the team training, which meant that no extra costs were required to educate instructors.

Missed revenues were included because the OR and the OR team could not be used for clinical purposes, as no clinical production was delivered by these teams during the training sessions.The hospital business administration office estimated missed revenues based on the revenues generated by different surgical specialties. This led to a range of costs, as different surgical specialties generate a different amount of revenue. The surgical specialties that were included in the calculation are: general surgery, trauma surgery, gastro-intestinal surgery, surgical oncology, vascular surgery, cardiothoracic surgery, otorhinolaryngology, oral and maxillofacial surgery, gynaecology, urology, orthopedic surgery, plastic and reconstructive surgery, neurosurgery, and ophthalmology.

The total cost of the team training was evaluated as a value per training session, in line with the “rental charge” as described by Maloney and Haines [13]. Furthermore, we determined the average number of participants for each training session, which enabled us to calculate the average cost of the training per participant.

Next, we considered the costs that had to be made to start with the team training, i.e., investment in a mannequin (Laerdal Resusci Anne and Difficult Airway trainer add-on) and simulation software (REALITi 360 patient simulator monitor, Secta Medical). The costs of this equipment are written off over 10 years. We have therefore used this timeframe to calculate the costs per session.

Lastly, we compare the costs of the simulation training to daily practice, i.e., a regular surgical program. The goal of the study was to calculate the costs of a CRM simulation training; however, we feel that it is also important to place these costs in perspective to ‘business-as-usual’. These costs were calculated by adding the costs for the instructors, materials, and missed revenues. Salaries and overhead costs were not included, as these costs are also made during regular work activities.

Cost was recorded in euros (EUR). For clarity, amounts were rounded toward the nearest whole number.

The ethical committee of the Dutch Society for Medical Education reviewed and approved a study we conducted on the effects of this team training program (NVMO, file number 2020.8.8). The current study is a follow-up study and uses additional data that were collected in relation to the original study. The newly collected data do not concern healthcare professionals or patients. Therefore, no additional ethical review was deemed necessary for this study.

## Results

Resources for the team training are listed in Table 1 (for the costs in dollars see Table S2 in the Supplementary material 1). The cost of participants (i.e., their salaries) accounts for between €220 and € 600 per person, per session, depending on the profession of the participant. Instructors also contribute to the costs, with external facilitators being more expensive than in-house trainers at €469 per session. In-house trainers cost on average €410, depending on which professional is available as a trainer. Materials, such as surgical supplies, add an additional €41, and the mannequin and simulation software resp. €33 and €90 per training session. For a complete breakdown of the material costs, see Table S3 in the Supplementary material 1. Overhead costs, including the use of the OR, amount to €800 for each session.

**Table 1:** Costs of the resources of the team training in euros

Resources	Costs per unit	Costs per training session of 4 h
Participants		
- 1 surgeon	€150/h	€600
- 2 operation assistants	€60/h/person	€480
- 1 anesthesiologist	€150/h	€600
- 1 anesthesia assistant	€55/h	€220
Instructors		
- External facilitator	€938/day	€469
- In-house trainer (anesthesiologist or anesthesia assistant)	€150/h or €55/h	€410 (mean)
Materials		
- Mannequin	€6.600/10 years	€33
- Software	€18.000/10 years	€90
- Supplies such as syringes, tubes, i.v. catheters, surgical instruments	€817/year	€41
Overhead (i.e., use of OR, light, gas, cleaning, management, maintenance of OR)	€200/h	€800
Missed revenues due to not using the OR (dependent on type of surgical specialty)	€2.000–€3.000/h	€8.000–€12.000
Total		€11.743–€15.743

Missed revenues due to not utilizing the OR and the surgical team range from €8.000–€12.000 per session, depending on the type of surgery that would have been performed. The total costs of a 4-h session add up to €11.743–€15.743. This leads to costs of €2.349-€3.149 per participant. Without the missed revenues, the costs are, respectively, €3743 per training session and €749 per participant. Participants’ salaries make up approximately 12 to 16% of the cost per training session.

The total costs of the simulation team training include both salaries and missed revenues. If you would compare the costs of simulation training to daily practice (i.e., an ordinary day of elective surgery), the costs of the training add up to €9.043–€13.043. The costs would mainly be attributed to missed revenues. Salaries and overhead costs were not included in this calculation, as these were also made during daily practice.

The described costs do not include implementation costs. If the training were still to be implemented, one should consider the initial investment costs for purchase of materials.

The costs for purchasing a mannequin and software amount to €24.600. In the Netherlands, the EuSim certification costs €2.000. Also, the costs of organisational meetings prior to implementing this training should be considered.

## Discussion

Providing in-situ interprofessional CRM training for surgical teams is a costly affair, especially from a hospital perspective, considering missed revenues. An OR is an expensive environment, where maintenance costs are high and where team members have relatively high salaries, compared to, e.g., a general ward. While the costs of setup of a team training program are relatively limited, repeated performance of these training sessions requires considerably more money. This study was conducted from a hospital administration perspective. However, from a societal perspective, it could be considered a responsibility of medical educationalists to consider how education programs impact the hospital and its funds. Evaluating and acknowledging expenses related to providing CRM training will help to make informed decisions about the best allocation of limited funds and time in healthcare education.

Only a few previous studies specifically evaluated the costs of a high-fidelity in-situ interprofessional team training program. Rosqvist and colleagues implemented a 2-h in-situ trauma team training in a Finnish emergency department [9]. They trained 81 teams across 124 simulations, at a cost of €58.000 per year, which breaks down to €203 per participant and €1.220 per team. They found a cost of €427 per point increase in teamwork performance on a 25-point scale. However, their training included six team members, compared to five in our training, and the duration was 2 h, compared to 4 h in our training session. Furthermore, the calculation did not account for lost revenues associated with the training, which means that the actual costs of their team training were likely higher than reported. Lost revenues for an ER team could be more difficult to evaluate than for an OR team. However, the situation is identical in a sense that during training the facilities and the participants are not available for clinical care. The same Finnish group also developed a 2-h simulation training concerning a ruptured abdominal aortic aneurysm [10]. They found that the total costs for 21 teams was €29.415, of which 20% were implementation costs and 80% were education costs (i.e., salaries, materials, maintenance of equipment and hire of rooms). They did not include missed revenues in their cost analysis. Furthermore, a Dutch research group evaluated the cost of a 1-day, simulation-based, multiprofessional obstetric team training program [8]. This program included repetition sessions of half a day, either in-situ or in a simulation center. The initial training session cost €25.546, while the repetition sessions cost €9.035, with an average of six participants per session. This brings the cost per participant for repetition sessions to approximately €1.506. Two other studies examine the costs associated with large-scale CRM implementations at the hospital or even country-wide level, which makes them difficult to compare to our training program [14, 15].

Comparing different interprofessional CRM training programs is therefore difficult. When comparing our training program with other programs, it seems more expensive. However, the duration and number of participants differ, and most importantly, other studies have not included missed revenues. We are of the opinion that costs due to missed revenues also need to be considered to determine the total costs of in-situ interprofessional CRM training, and we thereby provide a comprehensive overview of the total costs of such training programs.

### Possible benefits of CRM training

When considering positive effects, benefits related to team performance as well as benefits regarding the development and well-being of individual healthcare professionals can be relevant. Interprofessional CRM training has the potential to save costs by, for example, decreasing the occurrence of complications [15, 16]. It could also lead to increased work-related satisfaction and happiness in healthcare personnel [4]. Ideally, the expenses made in implementing a CRM training program should be put in perspective to these potential benefits. This benefit, however, is difficult to express in euros.

Theoretically, operating faster due to better teamwork can lead to lower costs, simply because the occupation of the OR is shorter or because, for example, fewer surgical site infections occur. It is as yet unclear whether CRM actually facilitates faster surgery [15, 16], but costs related to complications and repeat surgeries are substantial, running into the (tens of) thousands [17–19]. Also, length of stay increases in the case of complications. In this way, preventing complications will reduce hospital costs. In our hospital, a day of admittance to an ordinary ward costs €600–€800, which means that one training session is equal to 20 patient days (not considering aspects such as medication, examinations, surgeries, etc.).

Furthermore, interprofessional CRM training can help in building a healthy workforce and minimize the requirement of hiring temporary professionals [4]. In the short term, team training leads to a loss of revenues due to not performing surgeries. But this could also be considered an investment to train healthcare professionals and create a stable long-term workforce.

### Choices in team training design

It is also important to consider potential alternatives in the design of an interprofessional team training program. Our program was conducted in situ. Advantages are that the simulation is run in a familiar environment, in which actual patient care is also performed. The training helps prepare participants to manage emergency situations in their own workplace, with their own materials. Thus, in situ simulations increase the fidelity of the simulations [20]. Furthermore, situativity theory highlights how learners and their environment can interact, which influences the learning outcome [21]. Instructors can focus on the situations and the role that people play, due to the authenticity of the simulation environment, instead of the mental representation that learners have of the world [21]. Participants do not have to travel to an external site, thereby limiting the time needed for training. Also, in situ simulations test (a part of) the daily work systems, allowing exploration of potential deficiencies or defects [20].

However, performing in-situ simulations requires (expensive) materials and equipment and suitable space (although would be similar for off-site simulations). Furthermore, participants could potentially be disturbed by pagers or phone calls. However, off-site simulation training is not necessarily cheaper than in-situ training. The majority of costs consisted of loss of revenue, because the surgical team could not perform surgeries. This holds true for both in-situ and off-site training programs. A possible reduction in costs for not using OR facilities is easily offset by the costs of hiring an external simulation center to provide the training. An option that might be considered is to conduct in-situ simulations outside of regular working hours. This would minimize loss of revenue, although salaries would be higher, due to mandated bonuses for working out of hours.

Our in-situ team training consisted of high-fidelity interprofessional training. High-fidelity set-ups generally require more funds than low-fidelity; although studies indicate that low-fidelity simulations are generally not inferior to high-fidelity simulations [6, 22].

Also, other potential modalities for simulations could be considered. For example, table-top exercises, or the use of Virtual or Augmented Reality [23, 24]. This might be a less costly alternative, although effects on potential outcomes such as learning and team functioning need to be assessed.

Another relevant factor is the duration of the training. The goal is to create a training session that is efficient and has a long-term effect. The effect of a previous 1-day obstetric team training was shown to decline after 3 months, suggesting that repeated training is necessary to create a sustainable effect [25]. An evaluation of CRM training in the Netherlands indicated that the duration of CRM training varies from 4 to 15 h. CRM instructors were unanimous in stating that one session is insufficient for the full effect of training CRM principles [11]. It is still unclear what the optimal duration and interval between training sessions are, just as it is difficult to state the minimum level of CRM principles or non-technical skills participants should achieve. Further research is required on both these aspects.

Furthermore, it might also be beneficial for learners to observe simulations, instead of participating in simulations. This would lead to more participants per simulation training, possibly lowering the costs. However, both observers and participants in simulations would otherwise be engaged in work activities that generate revenue.

### Limitations

Our findings concern a team training in the Netherlands, a high-income country, which is probably not generalizable to surgical teams in other countries, especially not to teams in low- or middle-income countries. The type of simulation training differs between countries and the resources educators and clinicians have access to. Furthermore, the missed revenues also differ between hospitals, even within the Netherlands. A calculation such as ours needs to be adjusted to different institutions, but it (1) gives an example of costs that are included and can be used as a blueprint and (2) provides a notion of the magnitude of the costs involved. Also, in the Netherlands, healthcare institutions are (mostly) funded based on the output they generate. This might not be true for healthcare funding in other countries, which means that missed revenues are defined differently or are not as relevant.

Also, we had the advantage of pre-existing availability of certified instructors. This saved both costs and time in the preparation of the training program. If one would want to start from the beginning, this would lead to an increase of implementation costs.

We did not include possible beneficial effects of our intervention in this study, precluding a cost–benefit analysis. We do feel that it is necessary to see the costs of such a training in perspective to potential benefits to ascertain that this kind of training actually delivers value for money [26].

## Conclusions

We found that in-situ interprofessional team training is expensive, especially due to missed revenues of elective surgical procedures. These costs need to be transparent to enable educators and healthcare administrators to carefully allocate funds in their institutions. The funds needed for training sessions such as these could also be used for other initiatives to promote healthcare. It is therefore prudent to consider the design and effect of training programs, in order to make them as cost-effective as possible. In this paper we discuss that the costs of team training might balance against potential benefits, such as faster surgeries and creating a sustainable workforce. Future research should aim to compare (types of) simulations on both learner and patient outcomes and costs. Furthermore, it is important to include financial considerations in studies focusing on educational initiatives. Potentially high costs of training programs can be justified, if these programs are shown to improve learning (and preferably also patient care) in a significant way. But these costs should at least be known to make an informed decision on the balance between costs and benefits.

## Supplementary Information

Supplementary Material 1. Table S2: Costs of the resources of the team training in US dollars. Table S3: Costs for the materials per year in euros (20 training sessions).

## Data Availability

Data is provided within the manuscript or supplementary information files.

## Abbreviations

CRM: Crew resource management
ER: Emergency room
ICU: Intensive care unit
OR: Operating room
