# Supplementary material for: The surprising costs of on-site surgical team CRM training: a Dutch example analyzed
Source: Adv Simul (Lond). 2025 Jul 2;10:37. doi: 10.1186/s41077-025-00367-x (PMC12224696; doi:10.1186/s41077-025-00367-x)
Supplement: Supplementary file 1 — Supplementary Material 1. Table S2: Costs of the resources of the team training in US dollars. Table S3: Costs for the materials per year in euros (20 training sessions). [file 41077_2025_367_MOESM1_ESM.docx]

SUPPLEMENTARY MATERIAL

| Table 2: Costs of the resources of the team training in USD (converted from EUR) | | |
| --- | --- | --- |
| **Resources** | **Costs per unit** | **Costs per training session of 4 hours** |
| Participants   - 1 surgeon - 2 operation assistants - 1 anesthesiologist - 1 anesthesia assistant | $158/hour  $63/hour/person  $158/hour  $58/hour | $630  $504  $630  $231 |
| Instructors   - External facilitator - In-house trainer (anesthesiologist or anesthesia assistant) | $985/day  $158/hour or $58/hour | $492  $432 (mean) |
| Materials   - Mannequin - Software - Supplies such as syringes, tubes, i.v. catheters, surgical instruments | $6.930/10 years  $18.900/10 years  $858/year | $35  $95  $43 |
| Overhead (i.e. use of OR, light, oxygen, heating, cleaning, management, maintenance of OR) | $210/hour | $840 |
| Missed incomes due to not using the OR (dependent on type of surgical specialty) | $2.100-$3.150/hour | $8.400-$12.600 |
| **Total** |  | $12.332-$16.532 |
| *Currency Exchange Rate (CER) of 1 euro = 1.05 US dollar (CER December 12, 2024)* | | |

| Table 3: Costs for the material per year in euros (20 training sessions) | | | |
| --- | --- | --- | --- |
| **Materials** | **Amount required** | **Price per item** | **Costs** |
| Endotracheal tube | 20 | € 1,93 | € 38,60 |
| Oropharyngeal airway | 6 | € 0,39 | € 2,34 |
| Ventilation mask | 10 | € 1,34 | € 13,40 |
| Frova intubating introducer | 4 | € 47,48 | € 189,92 |
| Swivel | 20 | € 0,64 | € 12,80 |
| Filter | 20 | € 1,16 | € 23,20 |
| Laryngoscope blade (reusable) | 10 | € 1,00 | € 10,00 |
| Yankauer suction tip | 10 | € 0,86 | € 8,60 |
| Oxygen catheter | 10 | € 0,31 | € 3,10 |
| Oxygen masker | 6 | € 0,82 | € 4,92 |
| Gastric tube | 6 | € 5,35 | € 32,10 |
| IV catheter | 26 | € 0,64 | € 16,64 |
| Syringe 50cc | 10 | € 0,35 | € 3,50 |
| Syringe 10cc | 10 | € 0,06 | € 0,60 |
| Syringe 5cc | 10 | € 0,05 | € 0,50 |
| Syringe 2cc | 10 | € 0,04 | € 0,40 |
| Infusion pump line | 30 | € 0,22 | € 6,60 |
| Ringers lactate 500ml | 10 | € 0,65 | € 6,50 |
| Suture thread | 4 | € 1,29 | € 5,16 |
| (Sterile) surgical gown | 20 | € 2,94 | € 58,80 |
| (Sterile) split sheet | 10 | € 8,84 | € 88,40 |
| (Sterile) surgical drape | 10 | € 2,83 | € 28,30 |
| Blue OR table sheet | 8 | € 1,82 | € 14,56 |
| Transfer sheet | 8 | € 0,75 | € 6,00 |
| Disposable gloves | 100 | € 0,02 | € 2,00 |
| Surgical masks | 1 | € 0,11 | € 0,11 |
| Surgical hats | 1 | € 0,15 | € 0,15 |
| Medipore omnifix plaster | 5 | € 1 | € 5,00 |
| Gauze pack | 2 | € 0,49 | € 0,98 |
| Leukopor tape | 1 | € 0,77 | € 0,77 |
| Leukoplast tape | 1 | € 2,06 | € 2,06 |
| Big tegaderm plaster | 40 | € 1,25 | € 50,00 |
| IV dressing | 20 | € 0,31 | € 6,20 |
| Garbage bag | 10 | € 0,35 | € 3,50 |
| Cotton pad | 40 | € 0,18 | € 7,20 |
| Laparoscopic tray | 1 | € 29,75 | € 29,75 |
| Orthopaedic tray | 1 | € 70,07 | € 70,07 |
| Urology tray | 1 | € 25,75 | € 25,75 |
| Trauma tray | 1 | € 38,62 | € 38,62 |
| **Total cost per year: € 817,10** | | | |
